# Transgenerational skin adaptations to late-gestation heat stress in great-granddaughters

**DOI:** 10.3168/jdsc.2025-0786

**Published:** 2025-07-16

**Authors:** B.D. Davidson, K. Hardy, J. Laporta

**Affiliations:** Department of Animal and Dairy Sciences, University of Wisconsin–Madison, Madison, WI 53706

## Abstract

Homeostasis and thermoregulation depend on the interplay of the hair and skin. Maternal heat stress in late gestation triggers postnatal hair and skin adaptations in daughters and granddaughters. Herein, we investigated the transgenerational effects of late-gestation heat stress on the hair and skin of the great-granddaughters. Pregnant dams (F_0_) were heat stressed (shade) or cooled (shade, fans, water soakers) during the last ~56 d of gestation in a subtropical summer. The first generation of heifers (F_1_) that experienced in utero heat stress (HT_F1_) or cooling (CL_F1_) were managed as a cohort until they gave birth to the second generation of heifers (F_2_) that experienced heat stress (HT_F2_) or cooling (CL_F2_) as a germ cell within the F_1_ fetal ovaries. All F_2_ heifers were managed as a cohort and gave birth to the third generation of heifers (F_3_; great-granddaughters) that were unexposed to the F_0_ treatments (HT_F3_ and CL_F3_). At 70 d of age, a hair sample and skin biopsy were collected from the neck on a subset of F_3_ heifers (n = 6/group). Hair length and diameter; skin epidermis thickness and area; dermis thickness; sweat gland (SWTG) number, area, and depth; and sebaceous gland (SEBG) number, area, and depth were evaluated using hematoxylin and eosin staining and analyzed in ImageJ. Data were analyzed using PROC MIXED in SAS. No differences were captured in hair length or diameter, epidermis thickness or area, depth of the shallowest SWTG, depth of the SEBG, dermis thickness, and the number or size of SWTG. The HT_F3_ had fewer (13.3 vs. 17.8 ± 1.1 glands) and smaller SEBG (61,641 vs. 89,963 ± 6,768 µm^2^), relative to CL_F3_. Although SWTG number or size did not differ, distance from the skin surface to the deepest SWTG tended to be shorter in the HT_F3_ (1.1 vs. 1.3 ± 0.04 mm). Late-gestation heat stress alters SEBG size and number and SWTG localization of F_3_ heifers, suggesting a transgenerational programming of postnatal thermal adaptivity.

## Highlights

• Late-gestation maternal heat stress did not affect the hair coat of the F_3_.
• Great-granddam heat stress led to fewer and smaller sebaceous glands in the F_3_.
• Heifers from heat-stressed lineages have less skin coverage of sweat glands

## Graphical Abstract

**Figure:**
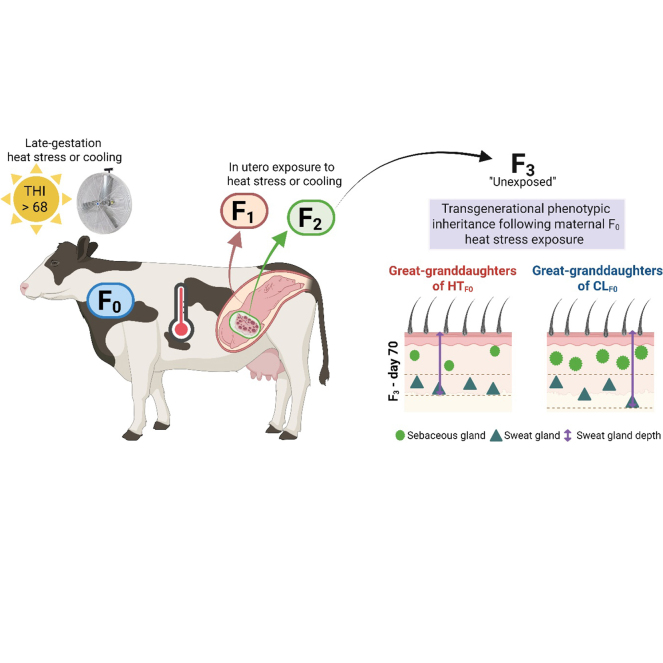
**Summary:** As homeotherms, cattle maintain their core body temperatures within a narrow range through means of evaporative cooling. The skin and hair coat are important for heat dissipation and thermoregulation in environments where temperatures and humidity are high. Late-gestation maternal (F_0_) heat stress has been shown to alter the daughter's (F_1_) and the granddaughter's (F_2_) hair length and diameter, in addition to the distribution of sebaceous and sweat glands through the skin layers. Herein, we report a transgenerational phenotypic inheritance of hair and skin characteristics in the third “unexposed” generation of dairy heifers. The F_3_ generation of heifers arising from the heat-stressed (HT) lineage have fewer and smaller-sized sebaceous glands and sweat glands that do not span as deeply in the skin, relative to the F_3_ heifers from the cooled (CL) lineages. These alterations in the skin properties of the third generation may affect their thermal adaptivity during their productive lives.

Current prediction models estimate that environmental temperatures could rise by 1.5°C to 2°C in 30 years, making temperatures of 52°C a possibility around the globe (IPCC, 2023). Rising temperatures pose welfare, sustainability, and productive threats to the dairy industry, as modern dairy cows are highly susceptible to heat stress (West, 1999; Collier et al., 2006; Das et al., 2016). As homeotherms, cattle maintain core body temperatures within a physiological range by balancing heat gain and heat loss (Mount, 1974; Hahn, 1999). The skin is the largest sensory organ and is a barrier between the internal and external environments, and hair characteristics act as an insulator with roles in thermoregulation (Romanovsky, 2014; Mota-Rojas et al., 2021). In hot environments, hairs flatten to reduce the amount of air trapped between the skin and hair to enhance heat loss and reduce body temperatures (Romanovsky, 2014).

Cattle become less effective at maintaining core body temperatures when the number of days and hours of heat stress exposure increase (Bernabucci et al., 2010). The rise in core body temperature of a pregnant cow influences the temperature of her fetus, and intrauterine stressors are known to influence the structural and functional roles of vital organs in the developing fetus, which may persist into adulthood (Barker, 1990; Desai and Hales, 1997; Asakura, 2004). The negative effects of maternal late-gestation heat stress on growth, immunity, organ development, and longevity of the resulting daughters are well-documented (Monteiro et al., 2016; Laporta et al., 2020; Ahmed et al., 2021; Dado-Senn et al., 2020). Although hair and skin begin developing in early gestation (~40–50 d), much of the maturation and functional programming occurs during late gestation. Because hair and skin are dynamic through the lifetime, the functional foundation established during prenatal development is critically important (Paus and Cotsarelis, 1999). Our group has previously reported multigenerational effects of maternal heat stress on hair and skin characteristics of dairy cattle. Negative outcomes for heifers exposed to in utero heat stress (**F_1_**) include altered skin and hair characteristics at birth, which persist through 1 yr of age. Specifically, in utero heat stressed heifers had longer hair, more and smaller sebaceous glands (**SEBG**), fewer and smaller sweat glands (**SWTG**), and reduced SWTG coverage in the skin (Davidson et al., 2022). Heifers (**F_2_**) exposed to in utero heat stress as a germ cell within the developing ovaries of the F_1_ experienced hair and skin adaptations that may confer superior thermal adaptivity. Specifically, the F_2_ heifers at 70 d of age had shorter and thicker hair, thinner skin, and more but smaller SEBG (Davidson et al., 2024).

Herein, we hypothesized that inheritance of hair and skin characteristics following late-gestation heat stress exposure are transgenerational, persisting beyond 3 generations. Although there is growing interest in the role of epigenetic inheritance in mammalian species, particularly livestock, the extent to which environmental insults, such as adverse intrauterine conditions, can induce heritable modifications remains unclear (Jirtle and Skinner, 2007). Given prior evidence that maternal (**F_0_**) exposure to heat stress alters skin and hair traits in the F_1_ and F_2_, the objective was to evaluate whether such phenotypes are retained in the third generation (**F_3_**) that was unexposed to the F_0_ treatments.

The Institutional Animal Care and Use Committee at the University of Wisconsin–Madison (protocol: A006602-A01) approved this portion of a longitudinal multigenerational study. The treatments and experimental design are outlined in Dado-Senn et al. (2021). Briefly, during the summer of 2020 at a commercial farm in Florida, pregnant Holsteins (F_0_, grand-dam, n = 82) were exposed to environmental heat stress (**HT_F0_**; shade of the barn, n = 41) or provided heat stress abatement (**CL_F0_**; shade, fans, and water soakers, n = 41), during the last 54 ± 5 d of gestation. The temperature-humidity index (**THI**) was calculated with the equation proposed for subtropical environments (NRC, 1971; Dikmen and Hansen, 2009) and remained above 68, indicating that all grand-dams (F_0_) were at risk of experiencing environmental heat stress during late gestation. However, grand-dams that were provided heat abatement had reduced respiration rates (−24 breaths per minute [**bpm**]; 77.4 vs. 53.5 bpm, HT_F0_ vs. CL_F0_, respectively) and skin temperatures (−1.9°C; 36.0°C vs. 34.1°C; Dado-Senn et al., 2021).

Offspring born to F_0_ grand-dams (F_1_, daughters, n = 73) experienced in utero heat stress (**HT_F1_**; n = 36) or not (**CL_F1_**; n = 37). The daughters were managed as a single cohort from birth until first calving (Dado-Senn et al., 2021; Davidson et al., 2022). In the Fall of 2022, the daughters gave birth to the granddaughters (F_2_, n = 30). Thus, the germlines, resulting in F_2_ granddaughters, were exposed to in utero heat stress (**HT_F2_**; n = 12) or not (**CL_F2_**; n = 18) through the fetal daughter's (F_1_) developing ovary during late gestation. The granddaughters were managed as a single cohort from birth until first calving of the great-granddaughters (F_3_, n = 20). These F_3_ heifers that were unexposed to the initial F_0_ treatments (**HT_F3_**; n = 9; **CL_F3_**; n = 11) were raised as a cohort through the preweaning period (July to December 2024) and exposed to an average ambient temperature of 13.2°C, average humidity of 76.3%, and average THI of 55.6 (data from the Wisconsin Environmental Mesonet station 5.3 km from the research farm).

Hair samples and skin tissue biopsies were obtained from the right side of the neck at 70 d of age from a subset (n = 6/group) of F_3_ heifers following procedures reported in Davidson et al. (2024). Briefly, hair was placed in plastic bags at room temperature until length analysis. Following procedures described by Sarlo Davila et al. (2019) and Davidson et al. (2022), hair was divided into short (undercoat) or long (topcoat) lengths. Ten hairs of each length were measured and an average length was calculated for all hairs, the undercoat, and the topcoat. The difference between the undercoat and topcoat lengths were calculated. Hair diameter was measured, and averages were calculated for all hairs, the undercoat, and the topcoat. To collect the skin biopsy tissue, a biopsy punch (Standard Biopsy Punch, 6 mm, Integra Miltex Life Sciences Corporation, York, PA) was used. The tissue was rinsed in PBS, fixed at room temperature in 10% neutral-buffered formalin, bisected, placed in histology cassettes, and stored in PBS at 4°C until further analysis. To visualize the skin histomorphology, the tissue was dehydrated, paraffin-embedded, sectioned (7 μm), fixed to glass slides, and stained with hematoxylin and eosin (Hematoxylin 7211, Clarifier 1, Bluing, and Eosin Y Alcoholic; Thermo Fisher Scientific). Slides were imaged using a Keyence BZ-X800 microscope (Keyence Corporation, Japan) at 4× magnification and cropped to 1,000 × 1,000 pixels. Pixels were converted to millimeters using the following conversion formula: 1,000 pixels = 2.145 mm. Morphological measurements of interest included epidermis thickness and cross-sectional area, dermis thickness, SWTG number, cross-sectional area, and depth, and SEBG number, cross-sectional area, and depth and were measured according to Davidson et al. (2022) with the ImageJ software (US National Institutes of Health, Bethesda, MD). The SWTG and SEBG depth was assessed at the top of the most superficial gland and the bottom of the deepest gland.

Statistical analyses were performed in SAS (version 9.4, SAS Institute Inc., Cary, NC). Residuals were visually evaluated for normality and tested using the Shapiro–Wilk statistic (UNIVARIATE procedure, SAS). Multiple covariance structures were tested, and the structure yielding the lowest Akaike information criterion was selected for each variable. Hair and skin measurements (i.e., epidermis thickness and cross-sectional area; dermis thickness; SWTG number, cross-sectional area, and depth; and SEBG number, cross-sectional area, and depth) were analyzed using a generalized linear mixed model with the main effect of the late-gestation maternal (F_0_) environmental treatment. Significance was declared at *P* ≤ 0.05 and tendencies at 0.10 ≥ *P* > 0.05. Data are presented as LSM ± SE.

At 70 d of age, the average length and average diameter of the hairs were not different between groups (all *P* ≥ 0.26; Table 1). Differences were not observed between HT_F3_ and CL_F3_ groups for epidermis thickness and area, dermis thickness, number and size of SWTG, depth of the most superficial SWTG, and both depths of the SEBG. However, the number (13 vs. 18 ± 1 glands; HT_F3_ vs. CL_F3,_ respectively; *P* = 0.02; Figure 1C) and cross-sectional area (61,641 vs. 89,963 ± 6,768.6 µm^2^; *P* = 0.01; Figure 1D) of SEBG were smaller in HT_F3_ heifers. Moreover, the distance between the skin surface and the deepest SWTG tended to be shorter in HT_F3_ heifers, relative to CL_F3_ heifers (1.15 vs. 1.25 ± 0.04 mm; *P* = 0.08; Figure 2F).

**Table 1:** Hair length and diameter of great-granddaughters (F_3_), whose great-granddams (F_0_) were exposed to late-gestation heat stress or provided heat stress abatement during a subtropical summer^1^

Variable^3^	Group		SEM	*P*-value^2^ F_0_ treatment
	Great-granddaughters of CL_F0_ dams (CL_F3_, n = 6)	Great-granddaughters of HT_F0_ dams (HT_F3_, n = 6)		
Hair length, mm				
Avg. length	16.35	18.25	1.26	0.31
Avg. undercoat	9.21	10.16	0.56	0.26
Avg. topcoat	23.48	26.34	2.11	0.36
Dif. S&L	14.27	16.18	1.79	0.47
Hair diameter, mm				
Avg. width	0.59	0.58	0.02	0.81
Short hair width	0.56	0.57	0.02	0.79
Long hair width	0.61	0.59	0.02	0.51

1 Pregnant dams (F_0_) were heat stressed (HT; shade of barn) or cooled (CL; shade, fans, and water soakers) for the last 54 ± 5 d of gestation. The first generation of heifers (F_1_) who experienced in utero heat stress, or not, gave birth to the second generation (F_2_) who experienced in utero heat stress, or not, as a germ cell within the F_1_ fetal ovaries. These F_2_ heifers gave birth to the third generation (F_3_; great-granddaughters) that were unexposed to the initial F_0_ treatments.

2 Significance was declared at *P*≤ 0.05 and tendencies at 0.10 ≥ *P* > 0.05.

3 Hair was collected from the neck of HT_F3_ and CL_F3_ at 70 d of age. Measurements included average length of all hairs (avg. length), average length of short hairs (avg. undercoat), average length of long hairs (avg. topcoat), difference between the length of the undercoat and topcoat (dif. S&L), average diameter of all hairs (avg. width), average diameter of short hairs (short hair width), and average diameter of long hairs (long hair width).

**Figure 1:**
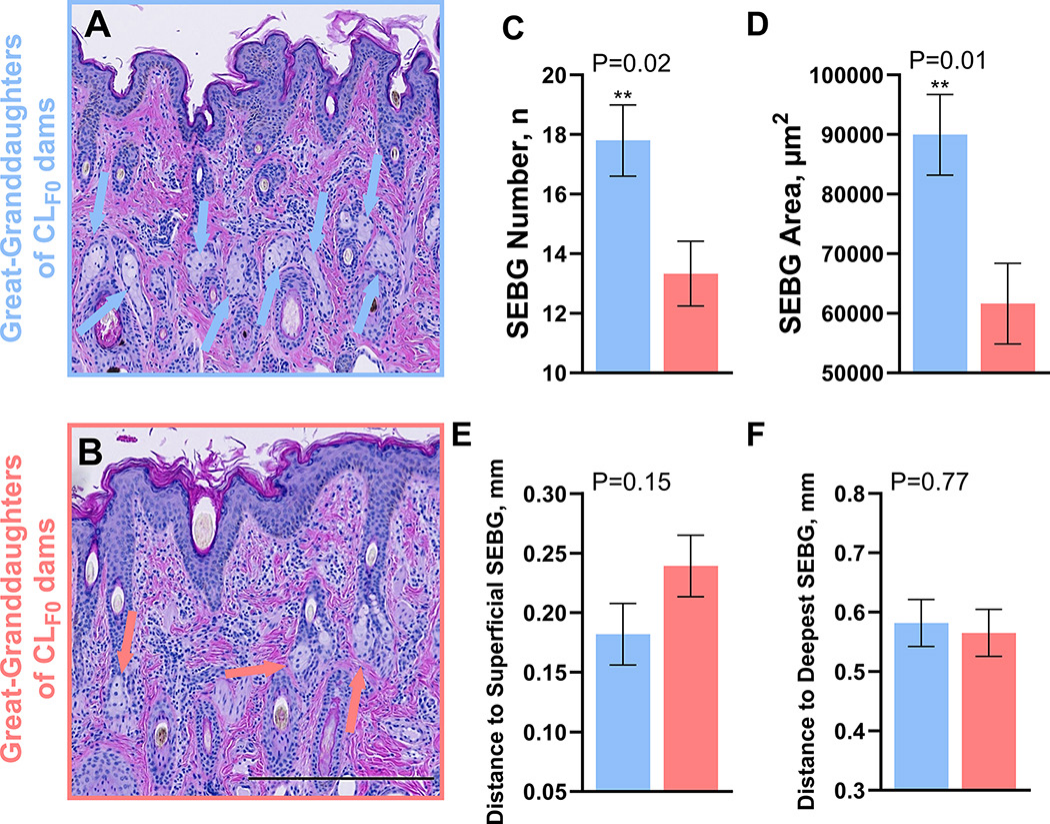
Sebaceous gland (SEBG) histomorphology of great-granddaughters at 70 d postnatal. Pregnant dams (F_0_) were heat stressed (HT; shade) or cooled (CL; shade, fans, and water soakers) for the last 54 ± 5 d of gestation. The first generation of heifers (F_1_), who experienced (or not) in utero heat stress, gave birth to the second generation (F_2_), who experienced (or not) in utero heat stress as a germ cell within the F_1_ fetal ovaries. The F_2_ heifers gave birth to the third generation (F_3_, great-granddaughters) that were unexposed to the initial F_0_ treatments. Skin tissue biopsies were collected from the neck of the great-granddaughters (HT_F3_ and CL_F3_, red and blue columns, respectively) at 70 d of age (n = 6/group) and stained with hematoxylin and eosin (A, B). Variables of interest included number of SEBG (C; indicated by the arrows in images A and B), cross-sectional area of SEBG (D), distance from skin surface to most superficial SEBG (E), and distance from skin surface to deepest SEBG (F). Data are presented as LSM ± SE. Significance was declared at *P* ≤ 0.05 (**). Microphotographs were taken at 10× magnification. Black scale bar = 500 pixels = 0.429 mm.

**Figure 2:**
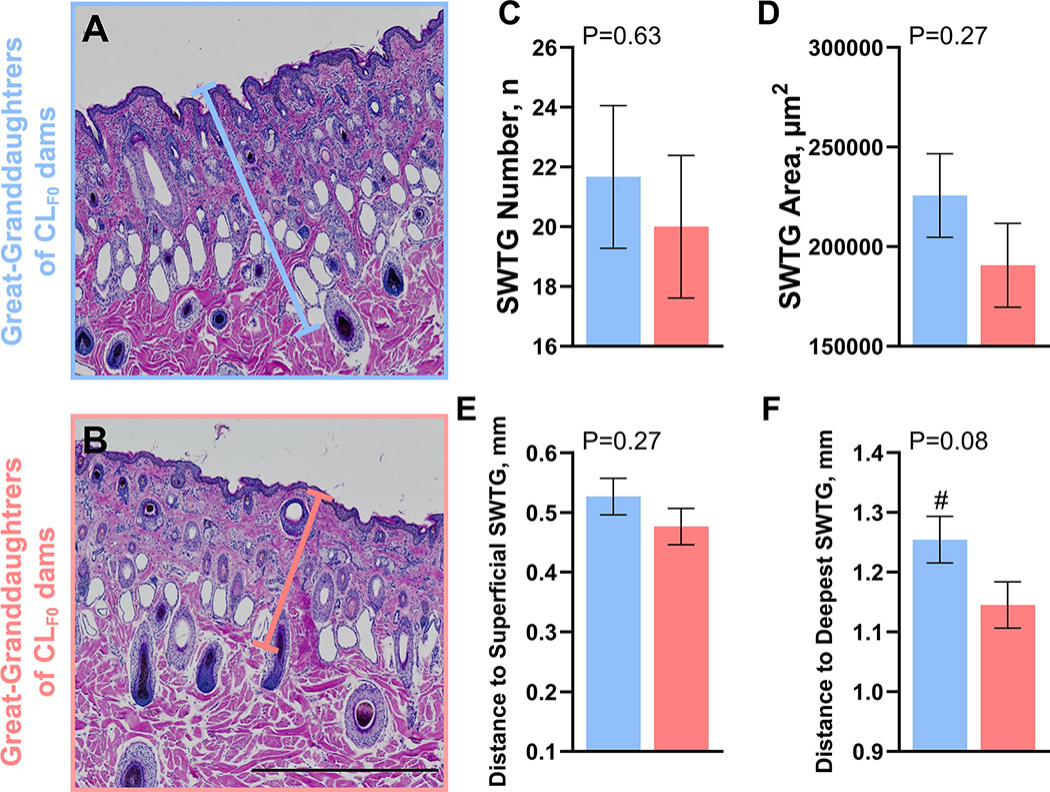
Sweat gland (SWTG) histomorphology of great-granddaughters at 70 d postnatal. Pregnant dams (F_0_) were heat stressed (HT; shade) or cooled (CL; shade, fans, and water soakers) for the last 54 ± 5 d of gestation. The first generation of heifers (F_1_), who experienced (or not) in utero heat stress, gave birth to the second generation (F_2_), who experienced (or not) in utero heat stress as a germ cell within the F_1_ fetal ovaries. These F_2_ heifers gave birth to the third generation (F_3_; great-granddaughters) that were unexposed to the initial F_0_ treatments. Skin tissue biopsies were collected from the neck of the great-granddaughters (HT_F3_ and CL_F3_; red and blue columns, respectively) at 70 d of age (n = 6/group) and stained with hematoxylin and eosin (A, B). Variables of interest included number of SWTG (C), cross-sectional area of SWTG (D), distance from skin surface to most superficial SWTG (E), and distance from skin surface to deepest SWTG (F; measurement depicted by colored lines in histology photos). Data are presented as LSM ± SE. Tendencies were declared at 0.10 ≥ *P* > 0.05 (#). Microphotographs were taken at 4× magnification. Black scale bar = 500 pixels = 1.0725 mm.

Adverse intrauterine conditions lead to structural and functional adaptations in the developing fetus. These alterations may not result in immediately observable phenotypes, but they can persist through maturity (Fowden et al., 2006; Skibiel et al., 2018). In utero heat stress is known to cause undesirable phenotypes, including reduced fertility, longevity, and production (Monteiro et al., 2016; Laporta et al., 2020). These phenotypes are determined by the interaction of genes and epigenetic modifications, including DNA methylation, histone modification, and RNA-mediated gene silencing (Landi et al., 2024). Growing evidence suggests that in utero heat stress exposure alters the epigenome of the offspring (F_1_; Skibiel et al., 2018) and the phenotypes of the second generation in dairy cows (F_2_; Laporta et al., 2020; Weller et al., 2021; Larsen and Laporta, 2024). However, animal studies documenting in utero exposure leading to epigenome modifications in the F_2_ are limited, with the most known research focusing on the agouti gene in mice and paternal methionine supplementation in sheep (Blewitt et al., 2006; Braz et al., 2022; Townsend et al., 2023). For phenotype changes resulting from heat stress exposure to be considered transgenerational in mammals, they must be present in the F_3_ generation that is unexposed to the F_0_ treatments (Khatib et al., 2024).

Herein, we investigated the transgenerational inheritance of hair and skin phenotypes in the F_3_ generation. Unlike our previous observations in the F_1_ and F_2_, no differences were observed between HT_F3_ and CL_F3_ for hair thickness or hair length. The HT_F1_ heifers had a shorter undercoat and a longer topcoat, traits that may compromise thermotolerance by entrapping hot air between the hair layers (Davidson et al., 2022). Additionally, HT_F2_ heifers had shorter and thicker hairs (Davidson et al., 2024). The hair coat plays an important role in insulating the skin and shorter hair provides a lower resistance to heat flow in white tailed deer (Jacobsen, 1980). Moreover, in Zebu cattle and cows with the slick gene, shorter, slicker, and less dense hairs provide them with a more efficient ability to regulate body temperature (Hansen, 2004; Dikmen et al., 2014). Although hair length is a determining factor in heat exchange, thermoregulation capacity is also affected by coat color, skin color and thickness, and hair follicle density (Collier and Gebremedhin, 2015). In cattle, one SEBG is associated with one hair follicle (Mateescu et al., 2023). In the current study, a reduction in SEBG number was observed in HT_F3_ heifers. Together, suggesting HT_F3_ heifers may have reduced hair follicle density, although this was not measured by our group. Although there were no differences in hair length and diameter, a possible reduction in the hair follicle density may suggest a programming of hair coat increasing thermotolerance in the F_3_ generation.

The lower number of SEBG in the HT_F3_ heifers aligns with findings from the HT_F1_ heifers at 63 d of age but contrasts with the findings for HT_F2_ heifers, who had an increased number of SEBG (Davidson et al., 2022, 2024). Although the thermoregulatory role of SEBG is not well understood in cattle, they are known to be highly innervated (Goodall and Yang, 1954; Jenkinson et al., 1966) and hormonally regulated (Thody and Shuster, 1989), and the sebum they produce minimizes skin moisture loss by discouraging sweat formation (Porter, 1993, 2001; Mateescu et al., 2023). Outside of SEBG number and size, Thody and Shuster (1989) suggest that their functional output is determined by proliferative activity, cell transition time, and lipid production. In the present study, the HT_F3_ heifers not only had fewer SEBG, but also had smaller sized SEBG. These observations may suggest that although HT_F3_ heifers could have equally functional SEBG as their cooled counterparts, the fewer and smaller sized glands, exhibiting a reduced coverage through the skin, may compromise optimal thermoregulation.

To maintain homeostasis through thermoregulatory means, cattle rely on various heat dissipation mechanisms, including evaporation from the skin surface, which is aided by sweat production (Johnson and Hales, 1983; Finch, 1986). In the current study, HT_F3_ heifers exhibited SWTG that were not as deep in the skin layers, spanning a smaller portion of the skin. These results are consistent with our previous observations in the F_1_, where HT_F1_ heifers had a reduced SWTG coverage within the skin (Davidson et al., 2022). However, current study results differ from our results in the F_2_, where no significant differences in SWTG morphology were detected between the HT_F2_ and CL_F2_ groups (Davidson et al., 2024). Although not assessed here, epigenetic modifications may be latent or subtle across generations, diminishing the likelihood of consistent phenotypic expression. This may also reflect a case of epigenetic skipping for specific hair and skin traits.

Similar to SEBG, the role of SWTG in thermoregulation is multifaceted. Sweating rate in cattle is influenced by the size, density, number, and the depth of the SWTG (Nay and Hayman, 1956). Although in the current study the SWTG coverage in the skin starts at the same deepness, the HT_F3_ heifers have SWTG that do not reach as deep in the skin. The distance to the most superficial SWTG appears to be more important for thermoregulation, as reported in thermotolerant Zebu cattle, which not only exhibit higher SWTG density but also SWTG localized closer to the skin surface (Dowling, 1955; Nay and Hayman, 1956). There is a positive relationship between SWTG size and depth with sweating activity (Nagib Nascimento et al., 2019) and although we do not capture size differences, SWTG that cover more of the skin layers may offer more control over temperature regulation.

In conclusion, F_3_ heifers arising from the heat-stressed lineage had reduced SEBG size and numbers, as well as SWTG that did not span as deep, occupying a smaller portion of the skin tissue. These skin characteristics, along with altered hair coat dynamics, are known to hinder thermoregulatory efficiency in cattle, leading to higher respiration rates, skin temperatures, and core body temperature in warm weather. Collectively, these findings support the concept that a brief period of late-gestation heat stress exposure can exert transgenerational effects on thermal adaptivity, likely through a programing of skin morphology in great-granddaughters. Future studies are needed to identify the molecular and epigenetic controls of these phenotypic alterations that lead to transgenerational thermal adaptivity in livestock.
